# Gynecological Cancer and Venous Thromboembolism: A Narrative Review to Increase Awareness and Improve Risk Assessment and Prevention

**DOI:** 10.3390/cancers16091769

**Published:** 2024-05-03

**Authors:** Anna Falanga, Domenica Lorusso, Nicoletta Colombo, Gennaro Cormio, Benilde Cosmi, Giuseppa Scandurra, Vanna Zanagnolo, Marco Marietta

**Affiliations:** 1 Department of Medicine and Surgery, University of Milan-Bicocca, 20900 Monza, Italy; anna.falanga@unimib.it (A.F.); nicoletta.colombo@ieo.it (N.C.); 2 Department of Immunohematology and Transfusion Medicine, Hospital Papa Giovanni XXIII, 24127 Bergamo, Italy; 3 Fondazione Policlinico Universitario A. Gemelli, Catholic University of Sacred Heart, 00168 Rome, Italy; 4 Gynecologic Oncology Program, European Institute of Oncology, IRCCS, 20141 Milan, Italy; 5 Gynecologic Oncology Unit, IRCCS Istituto Tumori “Giovanni Paolo II”, 70124 Bari, Italy; gennaro.cormio@uniba.it; 6 Department of Interdisciplinary Medicine (DIM), University “A. Moro”, 70124 Bari, Italy; 7 Angiology and Blood Coagulation Unit, Department of Medical and Surgical Sciences, University of Bologna, 40126 Bologna, Italy; benilde.cosmi@unibo.it; 8 Angiology and Blood Coagulation Unit, IRCCS Azienda Ospedaliero-Universitaria di Bologna, 40138 Bologna, Italy; 9 Unità Operativa Oncologia Medica, Ospedale Cannizzaro di Catania, 95126 Catania, Italy; giusy.scandurra@gmail.com; 10 IEO European Institute of Oncology, 20141 Milan, Italy; vanna.zanagnolo@ieo.it; 11 Hematology Unit, Azienda Ospedaliero-Universitaria, 41125 Modena, Italy; marco.marietta1@gmail.com

**Keywords:** gynecological cancer, venous thromboembolism, risk factors, risk assessment, pharmacological prophylaxis

## Abstract

**Simple Summary:**

Preventing venous thromboembolic complications in patient with gynecological cancers is a relevant issue and an unmet clinical need. Using a thorough literature search, we found that this problem is underestimated and preventative antithrombotic measures are underused. In addition, there are no specific validated risk assessment tools in this setting to help in clinical practice the identification of patients who are at highest thrombotic risk, who may most benefit from pharmacological anticoagulant prophylaxis, and avoid the anticoagulation-associated bleeding risk in those who are at low risk. We suggest that more research be done in this field to improve these patiens’ care.

**Abstract:**

The prevention and appropriate management of venous thromboembolism in cancer patients is of paramount importance. However, the literature data report an underestimation of this major problem in patients with gynecological cancers, with an inconsistent venous thromboembolism risk assessment and prophylaxis in this patient setting. This narrative review provides a comprehensive overview of the available evidence regarding the management of venous thromboembolism in cancer patients, focusing on the specific context of gynecological tumors, exploring the literature discussing risk factors, risk assessment, and pharmacological prophylaxis. We found that the current understanding and management of venous thromboembolism in gynecological malignancy is largely based on studies on solid cancers in general. Hence, further, larger, and well-designed research in this area is needed.

## 1. Introduction

The close relationship between venous thromboembolism and cancer has been recognized since 1865 in the landmark description by Armand Trousseau, to such an extent that malignancy has been defined as “a solid phase coagulopathy” [1,2]. Besides the epidemiological studies demonstrating the relationship between cancer and venous thromboembolism [3], a cause-and-effect relationship between in vivo thrombin generation, thrombosis, and cancer was rigorously established about 30 years ago by the seminal studies of Simon Karpatkin and his group [4].

Such a relationship has been further confirmed by a large amount of experimental and clinical studies. In particular, the available data show that cancer cells can release procoagulant factors, such as tissue factor, which initiate the coagulation cascade and promote thrombin generation; thrombin has been shown to promote tumor cell proliferation, angiogenesis, and migration, which are important processes in the growth and spread of cancer. Moreover, through both direct and indirect mechanisms, cancer cells can induce the expression of a procoagulant phenotype in monocytes, neutrophils, platelets, and endothelial cells, promoting the formation of blood clots [5,6,7,8].

The venous thromboembolism incidence in cancer patients has increased over the years because of different factors, including new therapies (e.g., angiogenesis inhibitors, immunotherapy, or hormonal therapies), improved survival, and high-resolution imaging, which allows for a timelier diagnosis of this complication [9]. After cancer itself, venous thromboembolism represents the second cause of death in patients with cancer [10,11]. Other than the substantial morbidity and mortality, venous thromboembolism in cancer patients may lead to withdrawal or delayed access to cancer treatments, long or recurrent hospitalization, contraindication to receiving some therapies (e.g., anti-angiogenics) and enrollment in clinical trials, and may account for significant psychosocial distress for patients and their caregivers, as well as for high healthcare system costs [12,13].

Current guidelines strongly recommend the use of pharmacologic prophylaxis of venous thromboembolism in hospitalized surgical and medical cancer patients, and suggest its usefulness also in selected ambulatory ones [14,15,16,17,18]. A careful and tailored preventive approach is recommended, and the extension of the prophylaxis also to discharged patients is suggested, as the management of established venous thromboembolism is particularly challenging in these subjects, being associated with a high risk of recurrence and anticoagulation-related bleeding [19,20]. However, despite this evidence, the literature data report an underestimation of this major health problem in cancer patients and an unsatisfactory use of venous thromboembolism prophylaxis in this patient setting [21]. Among the several factors contributing to this disappointing behavior, the absence of valuable clinical tools able to evaluate the specific and individualized risk of venous thromboembolism may play a major role. Accordingly, the authors of the recently published European Society of Medical Oncology Guidelines [17] strongly endorsed the development of cancer-specific risk assessment models to further refine current risk stratification approaches or to develop new models that incorporate promising biomarkers [22,23,24], given that thrombogenic potential varies depending on the type of cancer, the setting of disease, or the presence of certain oncogene mutations/rearrangements [25,26,27,28,29,30]. 

All of the above-mentioned considerations also apply to gynecological cancers. Although the literature data report an underestimation of venous thromboembolism in the gynecological setting, as for the general cancer patient population, an incidence of this complication up to 27% can be assumed [31,32,33,34,35,36]. This finding is not surprising, as many factors specific to gynecological neoplasms (i.e., the compression of pelvic veins by the tumor mass [37]) add to the general cancer-related prothrombotic state. Moreover, a decline or inconsistent use of prophylaxis in this setting has been reported [21,38,39]. A possible explanation for such an underestimation of the problem is that there are no risk assessment models for the stratification of individual venous thromboembolism risk that have been properly validated in the setting of gynecological cancer patients, despite all of the validated models for the stratification of individual risk in surgical and medical hospitalized patients including cancer as a risk factor.

On these grounds, raising awareness of the importance of the prevention and early detection of venous thromboembolism in gynecological cancer patients is considered a high priority, and the improvement and dissemination of information among physicians and healthcare professionals represents a current need in this setting. 

To this aim, this narrative review provides a comprehensive analysis of the current literature on the specific issue of venous thromboembolism in gynecological tumors, including ovarian, cervical, and endometrial cancers, by examining the risk factors, the risk assessment models, and pharmacological prophylaxis in this specific setting. 

## 2. Methods

A PubMed search was performed up to September 2023 according to the main topics of the review, i.e., risk factors and risk assessments of venous thromboembolism in gynecological cancer patients and pharmacological prophylaxis of venous thromboembolism in gynecological cancer patients. Different combinations of pertinent Medical Subject Headings (MeSHs) and free-text terms (e.g., gynecological cancer AND venous thromboembolism; gynecological cancer AND venous thromboembolism AND risk factors; gynecological cancer AND venous thromboembolism AND risk assessment; gynecological cancer AND venous thromboembolism AND prophylaxis) were used, focusing on papers published in English without a time restriction. Papers were selected for inclusion in this narrative review according to their relevance to the topic, as judged by the authors.

## 3. Venous Thromboembolism and Gynecological Cancer: Risk Factors

As for the general cancer population, and within the gynecological cancer cohort, some patient characteristics have been shown to be significant risk factors for venous thromboembolism, such as age, diabetes, and hypertension [33]. In particular, patients older than 60 years have been reported to have an increased risk of venous thromboembolism (Table 1) [40,41]. 

Although a high body mass index is generally considered a risk factor for the development of venous thromboembolism, and this parameter is included in most of the available risk assessments, studies specifically looking at patients with gynecological malignancy reported discordant data about the relevance of this factor [33,46,47]. 

A wide consensus is shared on the implication of specific gynecological tumor factors, including type, size, and stage in the risk profile of venous thromboembolism. In particular, it has been reported that the ovarian cancer population has a higher chance of experiencing venous thromboembolism than other gynecological tumor types, particularly within the first year after diagnosis (Table 1) [34,37,48,49,50]. This can be due to the tumor’s location in the pelvis, massive ascites compressing the major blood vessels, and procoagulant factors released by the tumor [37]. Moreover, more than 80% of women with ovarian cancer present with late diagnosis and metastatic disease at the exordium due to the lack of a screening tool for this cancer type. These factors contribute to the increased risk of venous thromboembolism, as the advanced stage is defined as an independent tumor-related risk factor (Table 1) [37,50]. Among ovarian tumors, clear-cell carcinomas have been associated with higher venous thromboembolism rates than other histologies (Table 1) [44,51,52]. Regarding cervical cancer, the dimensions of the tumor are correlated with an increased risk of venous thromboembolism: the >50 mm tumor size has a nine-fold increased risk (10% vs. 1%), as does the International Federation of Gynecology and Obstetrics stage IV (28% stage IV vs. 3% stages I–III) (Table 1). Moreover, the frequent presence of bulky lymph nodes in advanced stages further increases the venous thromboembolism risk due to lymphadenopathy-related pelvic vein compression [41,47,53]. Regarding endometrial cancer subjects, venous thromboembolism incidence has been correlated with tumor histology: endometrioid grade 3 histology, usually diagnosed in an advanced stage, has been associated with an increased prospect of a 6-month venous thromboembolism incidence compared with low-grade histologies (Table 1); moreover, endometrial cancer is also associated with hypertension, diabetes, obesity, and advanced age, all of which are venous thromboembolism risk factors [54]. 

Cancer-related risk factors also include genetic somatic alterations occurring after an aberrant mRNA transcription in an oncogene or tumor suppressor gene. In some cases, the resulting oncoprotein can lead to the extracellular secretion of cytokines and other proteins, with ensuing platelet and neutrophil activation and production of membrane-derived microparticles leading to a procoagulant phenotype. Tissue factor, a critical initiator of the coagulation cascade, represents the most investigated procoagulant effector in this cascade; its activity can be mediated by numerous oncogenes, such as *ALK*, *ROS1*, *HER2*, *KEAP*, *KRAS*, *STK11*, *EGFR*, *IDH1/2*, which can indirectly influence the coagulation cascade and thrombotic risk through several mechanisms (e.g., inflammation, angiogenesis) [55]. Literature evidence showed a relationship between tissue factor expression in ovarian cancer and venous thromboembolism, with tissue factor expression increased in clear-cell ovarian cancer and endometroid cancer, suggesting an explanation for the higher risk of venous thromboembolism in these subgroups [56,57,58,59].

Lastly, as for the general cancer population, the status of the tumor (active, in remission, or recurrence) has a role in the risk of venous thromboembolism. In particular, the presence of active cancer leads to a prothrombotic state due to various factors such as tumor-related inflammation, the release of procoagulant factors, and direct endothelial damage [60]. Patients with recurrent cancer face a renewed increase in venous thromboembolism risk, especially during periods of disease progression or aggressive treatment. Recurrence often involves the reactivation of tumor-related prothrombotic mechanisms, similar to those seen in patients with active cancer. 

### 3.1. Surgery

Surgery, integral to most treatment regimens for gynecologic cancers, is a well-described risk factor for venous thromboembolism [37,42,48]. It has been reported that venous thromboembolism occurs in 6–7% of patients with gynecologic cancer after surgery, despite prophylaxis, and that in patients undergoing gynecologic cancer surgery, the risk of pulmonary embolism is increased 14-fold compared with patients undergoing surgery for benign disease [61]. In particular, the duration of the operation time being over 180 min, the amount of bleeding and blood transfusions, the presence of ascites and disseminated tumors, as well as low levels of albumin before surgery have been described as risk factors for postoperative venous thromboembolism [33,47,62]. Laparotomic surgery has been associated with an increased risk of postoperative venous thromboembolism [45], while minimally invasive gynecologic surgery has been associated with a decreased risk of venous thromboembolism [37,63]. 

### 3.2. Anticancer Therapies

Chemotherapy represents the mainstay treatment for a broad range of malignancies, including gynecological cancers. Several chemotherapy agents have been associated with an increased risk of venous thromboembolism (Table 1) [64]. Of note, anti-angiogenetic drugs, namely the inhibitors of the vascular endothelial growth factor pathway, have shown an increase in both major bleeding and thromboembolism, limiting their use in patients carrying a higher risk of either adverse event [65,66].

At the same time, a significant underestimation of the burden of venous thromboembolism associated with some types of chemotherapy has been suggested, due to the suboptimal reporting of adverse events in oncological trials [67]. 

The mechanisms by which anticancer agents increase venous thromboembolism depend on the pharmacology of the treatment itself and can involve endothelial injury, decreased anticoagulants, or increased procoagulants, leading to the activation of coagulation or platelets [64]. 

It should be stressed that, from an epidemiological point of view, a risk factor is defined as a variable associated with an increased risk of disease. However, as nicely addressed by Huitfield [68], there can be several definitions of risk factor, each of them more precisely describing a different relation between the dependent and the independent variable. The risk factors for developing VTE in cancer patients deal with the prognostic aspect and can be better defined as “any personal attribute that can be used to make more reliable predictions about future risk of medical conditions”. Accordingly, they do not comply with the definition of “treatment effect risk factors”, i.e., an action that may be taken to increase or decrease the probability of the outcome. 

Therefore, the assessment of a variable as a prognostic risk factor should only be regarded as a conceptual aid to make more evidence-based clinical decisions. 

## 4. Thromboembolic Risk Assessment in Cancer Patients

The ability to identify cancer patients at higher risk for venous thromboembolism would allow for the proper stratification of patients in prophylactic therapy. In order to reliably assess the individual patient’s venous thromboembolism risk, several risk assessment models have been developed and validated in different settings of care [69,70]. Although some of these have not been developed specifically in the oncology setting, they are also widely used for oncological patients. Below we briefly review the most widely utilized risk assessment models in surgical, medical hospitalized, and ambulatory cancer patients. Afterward, we describe the available risk assessment models specifically targeting gynecological cancer subjects. 

### 4.1. Risk Assessment Models for the Assessment of Venous Thromboembolism in Cancer Patients

The Padua prediction score is a 20-point risk assessment model including 11 items, developed for hospitalized medical patients by integrating additional empirically gained risk factors with the Kucher model (Table 2) [71,72]. The presence of a cancer diagnosis accounts for 3 points. According to the Padua prediction score, a 32-fold increased venous thromboembolism risk was correlated with a high score (≥4 points) in patients without prophylaxis compared with patients with a low score [71]. However, a subsequent study did not find a correlation between the risk categories and an incidence of venous thromboembolism, but with in-hospital death, suggesting that this model can be considered a general co-morbidity and disease severity index [73].

The International Medical Prevention Registry on Venous Thromboembolism (IMPROVE) venous thromboembolism risk assessment model was derived from a large international registry of hospitalized, acutely ill medical patients and consists of seven independent venous thromboembolism risk factors that are given 1–3 points each according to their strength of association with venous thromboembolism risk (Table 2) [74]. Applied thresholds suggest an increased risk if the cumulative score is ≥3 [75] or ≥4 points [76].

The Comparison of Methods for Thromboembolic Risk Assessment with Clinical Perceptions and AwareneSS in Real Life Patients-Cancer Associated Thrombosis (COMPASS-CAT) model focuses on patients with breast, lung, colon, or ovarian cancer and combines cancer-related with patient-related risk factors, indicating a high risk for venous thromboembolism for scores ≥7 points (Table 2) [77]. More recently, an additional model was validated by Pabinger and collaborators in two independent prospective cohorts in the Vienna Cancer and Thrombosis Study, providing a nomogram risk estimation model (Vienna-CATS nomogram score) including tumor site category and one biomarker (D-dimer) (Table 2) [78]. The new Vienna-CATS prediction score was recently validated in a large prospective cohort of metastatic cancer outpatients during chemotherapy, providing a moderately reliable model to discriminate between patients at low and high risk of venous thromboembolism (c-index 0.66; 95% CI 0.63–0.67) [79].

**Table 2:** Venous thromboembolism risk assessment scales used in gynecological cancer patients according to the different patient settings.

Scale Name	Risk Factors	Risk Groups	Ref.
**Hospitalized Patients**			
Padua prediction score	Cancer (3 points) Previous venous thromboembolism (3 points) Reduced mobility (2 points) Thrombophilia (2 points) Bedrest >3 days Recent trauma or surgery ≥70 years Heart or respiratory failure Acute myocardial infarction or ischemic stroke Acute infection Ongoing hormonal therapy	<4 points: Low risk ≥4 points: High risk	[71]
IMPROVE VTE RAM	Cancer Previous venous thromboembolism Age > 60 years Thrombophilia Current lower limb paralysis Bedrest > 7 days ICU/CCU stay	≥3 points: High risk [74] ≥4 points: High risk [75]	[74]
Prospective Comparison of Methods for thromboembolic risk assessment with clinical Perceptions and AwareneSS in real-life patients-Cancer-Associated Thrombosis (COMPASS-CAT) score	Antihormonal therapy/anthracycline treatment Cancer diagnosis ≤ 6 months Central venous catheter Advanced stage CV risk factors Personal venous thromboembolism story Platelet > 350 10^9^/L	0–6 points: Low/intermediate risk ≥7 points: High risk	[77]
Vienna Cancer and Thrombosis Study (Vienna-CATS)	D-dimer Tumor site risk	≥7 points: High risk	[78]
**Surgical Patients**			
Caprini risk score	Approximately 40 risk factors including: Age: >75 years as high-risk factor Type of surgery BMI ≥ 35 kg/m^2^ Family history of thrombosis Congenital or acquired thrombophilia Multiple trauma or spinal cord injury	1 point: Low risk 2 points: Moderate risk 3–4 points: High risk 5 points: Very high risk	[80]
**Ambulatory Patients**			
ONKOTEV Risk Prediction Model	Khorana score > 2 Metastatic disease Vascular or lymphatic compression Previous venous thromboembolism event	0 points: Low risk >2 points: Very high risk	[81]
ClinicalRAM for cancer-associated VTE	Cancer stage Treatment History of venous thromboembolism History of paralysis/immobility Recent hospitalization Asian Pacific Islander race (considered as venous thromboembolism risk)	Six risk categories: 0–2 points: Low risk 3–5 points: High risk	[82]
ONCOTHROMB score	Genetic risk score Body mass index > 25 Tumor type Tumor stage	Weighting of variables from the multivariate analyses and information on how to calculate the ONCOTHROMB score are lacking up to date	[22]
Khorana RAM score	Original tumor site: high risk for stomach and pancreas Pre-chemotherapy platelet count ≥ 350,000 mm^3^ Pre-chemotherapy white blood cell count > 11,000 mm^3^ Hemoglobin levels < 10 g/dL or use of erythropoietin Body mass index ≥ 35 kg/m^2^	0 points: Low risk 1–2 points: Moderate risk 3 points: High risk	[83]
**Gynecological Cancer Patients**			
Nomogram model to predict the VTE risk aftersurgery in patients with gynecological tumors	Age D-dimer Body mass index > 30 Surgical approach	See Figure 1	[42]
Nomogram model to predict the probability of VTE in patients with epithelial ovarian cancer	Age D-dimer PR IHC positivity Ki-67 IHC positivity	See Figure 2	[84]
Thrombogyn score for patients undergoing surgery and chemotherapy	Hemoglobin levels < 11.5 g/dL Body mass index ≥ 30 kg/m^2^ Chemotherapy	0 points: Low risk 1 point: Moderate risk 2–3 points: High risk	[85]

The Caprini risk score was developed in surgical patients and includes approximately 40 risk factors, marked by scores of 1–5 according to the disease burden (Table 2) [80]. A present or previous malignancy accounts for 2 points. Patients can be classified as low, moderate, high, or very high risk according to the obtained cumulative score (Table 2) [80]. Although the Caprini score is the recommended risk assessment model for surgical patients, it is also widely used in medical ones. There is still a lack of proper validation of the Caprini score in many patient populations and clinical situations, and a recently published paper showed that the thresholds of the Caprini score associated with increased risk of venous thromboembolism may vary across different specialties within a score range of 7–11 [86]. Of note, among 68 studies that enrolled more than 4 million patients, only one was conducted in a mixed gynecological and urological surgical population of 783 patients. Therefore, the results of this otherwise interesting systematic review can hardly be transferred to the specific population of gynecological surgical patients, which in other studies seemed to carry a low thrombotic risk in the clinical practice despite more than >90% of patients being assessed as high risk according to this risk assessment model [37,68].

The ONKOTEV Risk Prediction Model is a four-variable risk assessment model for ambulatory patients consisting of a Khorana score > 2, metastatic disease, vascular or lymphatic compression, and a previous venous thromboembolism event, and was validated in an independent prospective cohort [81,87]. A score > 2 is associated with a very high venous thromboembolism risk (Table 2) [81].

A further model was developed very recently by the Harris Health System and externally validated by the Veterans Affairs healthcare system [82], and then externally validated by MD Anderson Cancer Center Tumor Registry [88]. The score considered specific factors related to cancer stage and risks associated with treatment, as well as predictors of venous thromboembolism, such as the history of venous thromboembolism, history of paralysis/immobility, recent hospitalization, and Asian Pacific Islander race (conferring a decreased venous thromboembolism risk). Six risk categories can be obtained on the basis of the total score, which can be dichotomized into low risk (score 0, 1 or 2) or high risk (3, 4 or 5) (Table 2) [82]. This model had improved performance over the Khorana score (c-statistic, 0.71 vs. 0.65, respectively, for the new risk assessment model and Khorana score) and doubled the number of venous thromboembolism events in the high-risk stratum [82].

Another recently developed ONCOTHROMB score considered a newly defined genetic risk score, including nine genetic variants independently associated with venous thromboembolism, for the prediction of venous thromboembolism, along with body mass index > 25, tumor type, and tumor stage (Table 2) [22]. A good predictive capacity (AUC = 0.781, 95% CI: 0.735–0.822) was reported; however, it should be noted that tumor types were unequally represented in the derivation and validation cohorts, with 41% of patients having colorectal cancer and 48% with lung cancer, respectively, in the derivation and validation cohorts [22].

Despite their usefulness as a tool to drive and standardize the individual patient’s thromboembolic risk assessment, overall, the predictive power of the reported risk assessment models is low, with reported c-statistic values around 0.6 [70].

The Khorana risk assessment model score has been developed for ambulatory cancer patients following febrile neutropenia and other complications with the new chemo regimen, and in which venous thromboembolism was not a predefined outcome. It includes five risk factors: the original tumor site, pre-chemotherapy platelet and white blood cell count, hemoglobin levels, the use of erythropoietin, and body mass index (Table 2) [83]. According to the rating scale, patients are divided into three risk groups (Table 2) [83]. Although the Khorana score has been extensively validated, and almost all the most recent guidelines quote its use, its main limitation is represented by the dependence on the tumor site [43,83,89]. For instance, a very recent study suggested that the Khorana score is not effective in the risk stratification of patients with gynecological cancers [89]. Thus, novel risk assessment models have been developed in the past few years and externally validated for assessing venous thromboembolism risk in ambulatory patients with solid cancers (Table 2).

### 4.2. Venous Thromboembolism Risk Assessment in Gynecological Cancer Patients

Given that the Khorana score places all patients with gynecological cancer in the intermediate-risk category, it is of limited value in this population, as it does not adequately identify lower-risk patients [37,43]. Moreover, in all the above-reported studies that developed and validated risk assessment models, gynecological cancer patients were scarcely represented, accounting for less than 10% of the whole study population. This bias strongly limited the usefulness of these risk assessment models in the specific setting of gynecologic oncology and prompted the development of risk assessment models specifically targeting this population.

**Figure 1:**
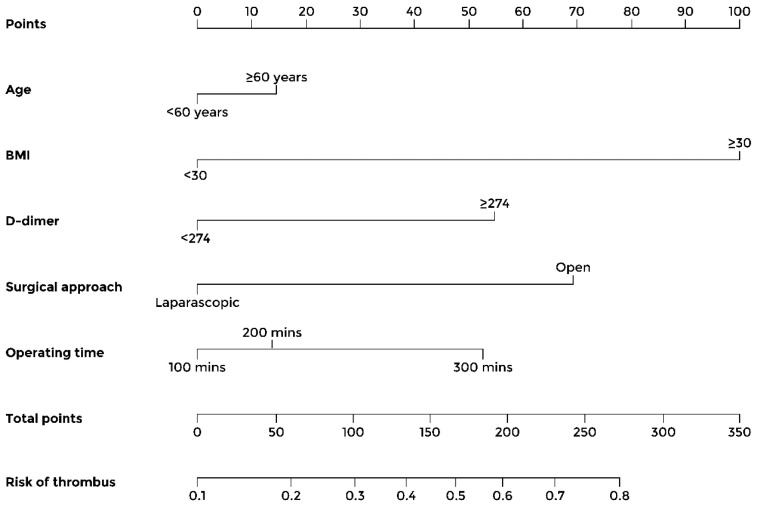
Nomogram predicting the post-surgical venous thromboembolism risk in gynecological cancers. Risk factors need to be referred to the point caliper (first row). The obtained points need to be added; the resulting sum can then be referred to the last row to obtain the predicted risk of venous thromboembolism. Reproduced with permission from [42] under a Creative Commons Attribution-NonCommercial 4.0 License.

**Figure 2:**
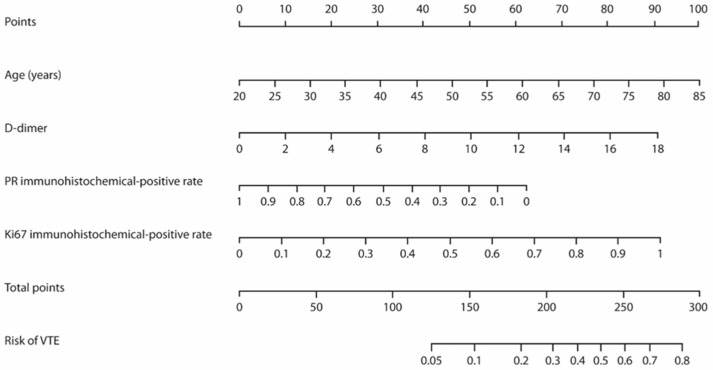
Nomogram predicting the venous thromboembolism risk in patients with epithelial ovarian cancer. Risk factors need to be referred to the point caliper (first row). The obtained points need to be added; the resulting sum can then be referred to the last row to obtain the predicted risk of venous thromboembolism. Reproduced with permission from [85] under a Creative Commons Attribution-NonCommercial 4.0 License.

In 2020, Wang and collaborators developed and validated a nomogram model based on five risk factors to predict the risk of venous thromboembolism in patients with gynecological malignancies. The included parameters were age, D-dimer value, body mass index, and surgical approach. The c-index of the model was 0.721 (95% CI: 0.6–0.7), with good discrimination and calibration effect (Table 2, Figure 1) [42]. In 2022, the same group developed an additional nomogram to predict the probability of venous thromboembolism specifically in patients with epithelial ovarian cancer, including progesterone receptor and Ki-67 immunohistochemistry positivity among predictors [85]. Though the AUC and calibration curve showed that the nomogram has a high accuracy, it still needs external validation to evaluate its prediction ability, as well as a multicenter validation with a larger sample size (Table 2, Figure 2) [85].

The Thrombogyn score has been developed as a risk model specific for gynecological cancer patients undergoing surgery and chemotherapy [84]. The variables included are body mass index, chemotherapy treatment, and hemoglobin levels, by which patients are categorized into three risk groups (Table 2) [22]. The Thrombogyn score performed adequately in both the derivation and validation cohort (0.714, 95% CI: 0.645–0.780 and 0.699, 95% CI: 0.605–0.792, respectively). The extension of this score with procoagulant-based biomarkers further improved its predictive value [84]. However, it has to be observed that the Thrombogyn score has not been externally validated.

The usefulness and limits of the VTE risk assessment models (VTE RAMs) are worthy of further consideration. RAMs are developed to estimate the probability of having a certain outcome (e.g., disease, event, or complication) in an individual, given its demographics, test results, or disease characteristics. The probability estimates can guide care providers as well as the individuals themselves in deciding upon further management, but they do not encompass every aspect of the clinical issue.

Moreover, no randomized, interventional study has assessed the performance of VTE RAM to steer the decision to provide pharmacological prophylaxis or not in every single patient. Therefore, probabilities estimated by a prediction model are not considered to replace but rather help the doctor’s decision making, serving as a useful tool to incorporate all the single pieces of information to aid clinical reasoning.

## 5. Venous Thromboembolism Pharmacological Prophylaxis in Cancer Patients

Given the high baseline venous thromboembolism risk, primary thromboprophylaxis with low-molecular-weight heparin is indicated in most medical and surgical hospitalized cancer patients, including gynecological ones. Furthermore, a growing proportion of cancer outpatients are candidates for primary prophylaxis with low-molecular-weight heparin or specific direct oral anticoagulants (apixaban or rivaroxaban), according to stratification scores [17].

It has been reported that these interventions can reduce venous thromboembolism incidence by 50–70% [90]. In clinical practice, guidelines and recommendations for venous thromboembolism prevention in the general cancer population apply to gynecological cancer patients. Table 3 summarizes the main guidelines and recommendations for venous thromboembolism prevention, according to cancer patient groups.

## 6. Venous Thromboembolism Prophylaxis in Hospitalized Cancer Patients

Hospitalized cancer patients present twice the risk of venous thromboembolism with respect to the general population, with an increased specific risk in gynecological cancer patients, as previously reported [34,90].

The Italian Association of Medical Oncology [91], the American Society of Clinical Oncology [15,93], the National Comprehensive Cancer Network [92], the European Society of Medical Oncology [17] and the International Initiative on Thrombosis and Cancer [16] guidelines strongly suggest the use of venous thromboembolism pharmacological prophylaxis with low-molecular-weight heparin, unfractionated heparin, or fondaparinux (factor Xa inhibitor) in all hospitalized cancer patients (Table 3). In particular, venous thromboembolism prophylaxis with low-molecular-weight heparin in hospitalized patients is highly recommended by the Italian Association of Medical Oncology and European Society of Medical Oncology [17,91]. The American Society of Clinical Oncology and NCCN guidelines suggest continuing venous thromboembolism prophylaxis in selected high-risk patients according to the Khorana score for up to 3–6 months after hospital discharge (Table 3) [40].

Low-molecular-weight heparins represent the first-choice agents for venous thromboembolism prophylaxis in cancer patients hospitalized for an acute medical illness [17]. However, some studies suggest that prophylaxis in this setting may not be appropriately targeted [94,95]. Thus, optimal pharmacological prophylaxis in cancer patients hospitalized for acute medical illness has yet to be defined.

## 7. Venous Thromboembolism Prophylaxis in Surgical Cancer Patients

Given that all cancer patients are classified at high thromboembolic risk, clinical practice guidelines by the Italian Association of Medical Oncology [91], American Society of Clinical Oncology [15,93], European Society of Medical Oncology [17], National Comprehensive Cancer Network [92], and the International Initiative on Thrombosis and Cancer [16] recommended pharmacologic thromboprophylaxis with unfractionated heparin or low-molecular-weight heparin, unless there is a contraindication related to a high bleeding risk, for all patients undergoing major surgery. The duration of postoperative thromboprophylaxis should be at least 10 days, but in selected patients, postoperative prophylaxis can be extended beyond this period. Indeed, evidence from meta-analyses has shown that extended thromboprophylaxis with low-molecular-weight heparin after major abdominal or pelvic cancer surgery reduces the risk of venous thromboembolism compared with a conventional duration of 2 weeks or less, without increasing the risk of major bleeding [96,97].

In particular, as shown by Guo and collaborators, in patients with cancer, the extended prophylaxis with low-molecular-weight heparin decreased the rates of venous thromboembolism (relative risk [RR] 0.20, 95% CI: 0.07–0.61) and pulmonary embolism (RR 0.13, 95% CI: 0.01–2.25) in comparison with no or mechanical prophylaxis, but potentially increased the risk of major bleeding (RR: 2.47, 95% CI: 0.08–74.18) [96]. This effect was not limited to open surgery but also occurred with some types of laparoscopic surgery, such as that for colorectal cancer [15,16,17,89,98].

At the same time, the quality of evidence derived from two clinical trials on the safety and efficacy of direct factor Xa inhibitors for extended postoperative thromboprophylaxis was deemed low in a very recent update of the American Society of Clinical Oncology guidelines, resulting in a weak recommendation regarding the use of apixaban or rivaroxaban, in addition to a prophylactic dose of low-molecular-weight heparin, after cancer surgery for patients who are candidates for extended prophylaxis [93,99,100].

Although these findings demonstrate that prophylaxis results were effective in surgical cancer patients, the optimal duration of this intervention is still unclear [97,101,102].

With specific regard to gynecological cancers, to date, three systematic reviews tried to assess the most effective and safe prophylaxis for perioperative and postoperative venous thromboembolism [40,103,104].

Einstein and collaborators used a direct meta-analysis to evaluate the effects of unfractionated heparin vs. low-molecular-weight heparin and unfractionated heparin vs. no prophylaxis on the risk of venous thromboembolism [103]. They found that unfractionated heparin could significantly prevent deep vein thrombosis by 42% relative to no prophylaxis, but no difference was reported with respect to low-molecular-weight heparin [103]. The systematic review by Rahn and collaborators included nine studies dealing with gynecological cancers, but they failed to apply a meta-analysis due to insufficient data [104]. The results showed that the overall incidence of clinical venous thromboembolism in gynecological cancers ranged from 0 to 15%, not significantly different from the 35% reported in patients who did not receive prophylaxis [104].

The most recent meta-analysis assessed the composite venous thromboembolism occurrence and major bleeding [40]. The reported results suggested that a combination of mechanical methods plus low-molecular-weight heparin was the best method to reduce composite venous thromboembolism occurrence, while sequential compression devices were the safest approach for major bleeding. However, a combination of sequential compression devices plus low-molecular-weight heparin provided the optimal balance between composite venous thromboembolism occurrence and major bleeding [40].

Moreover, different studies suggested that extended prophylaxis is not necessary for all patients with gynecologic cancers, particularly in those undergoing minimally invasive interventions [105,106,107].

## 8. Venous Thromboembolism Prophylaxis in Ambulatory Cancer Outpatients on Systemic Therapy or Carrying Central Venous Catheter

Cancer patients receiving systemic treatment are among the higher-risk populations for thromboembolic complications. High-prothrombotic agents include platinum compounds, 5-fluorouracil, capecitabine, gemcitabine, hormonal therapy, and anti-angiogenesis treatments, such as bevacizumab [108]. All the above-mentioned guidelines suggest medical prophylaxis in ambulatory outpatients under systemic therapy if considered at a high risk based on Khorana (>2 score) or other venous thromboembolism risk scores (Table 2) [15,16,17,91,92]. For instance, most recent guidelines also recommend the use of direct oral anticoagulants for the primary prophylaxis of VTE in cancer patients. However, DOACs are not authorized for this use in the EU. A recently published systematic review and meta-analysis demonstrated that among ambulatory cancer patients with an intermediate- to high-risk Khorana score of ≥2, thromboprophylaxis with direct oral anticoagulants or low-molecular-weight heparin can reduce the venous thromboembolism risk (number needed to treat, 25) without inducing an increased risk of major bleeding (number-needed-to-harm, 1000). Compared with low-molecular-weight heparin studies, the major bleeding risk seemed higher in direct oral anticoagulant studies (number-needed-to-harm, 100) [109]. However, it must be considered that gynecological cancer patients who are more likely to receive platinum-based therapies or anti-angiogenesis treatment have a higher risk of venous thromboembolism complications (Table 1) [110,111]. Accordingly, real-life evidence suggests considering the overall patient profile instead of solely the Khorana risk score in the risk assessment of venous thromboembolism [108].

Although in past years thromboembolic prophylaxis with low-molecular-weight heparin or warfarin in patients with central venous catheter was suggested, in the most recent guidelines, the medical prophylaxis was not routinely indicated in this patient setting (Table 3).

Some guidelines suggested the insertion of a central venous catheter on the right side and using a port instead of central venous catheters peripherally inserted to reduce central venous catheter–venous thromboembolism-related complications [83]. The discrepancy between the old and the new guidelines may be explained by introducing new catheters manufactured with less thrombogenic materials and the improvement of insertion techniques, which lead to a reduction in the risk of venous thromboembolism. However, in patients in whom thrombosis associated with central venous catheter occurs (estimated in about 2–7%), an anticoagulant therapy lasting at least 3 months is indicated, with a preference for using low-molecular-weight heparin [91].

## 9. Expert Opinion

The pathophysiology of venous thromboembolism in gynecological cancers is complex, and venous thromboembolism risk varies according to different factors [33]. Due to its debilitating and poor prognostic effect, the prompt prevention and management of venous thromboembolism in gynecological malignancy is important. However, the literature data report an underestimation of this major health problem in patients with gynecological cancers and a decline or inconsistent venous thromboembolism risk assessment and prophylaxis in this setting [12,13].

Therefore, a deep understanding of these aspects may help a proper venous thromboembolism risk stratification among gynecological cancer patients to identify which patients may benefit from different types and durations of prophylaxis.

The most recent guidelines suggest prophylaxis for surgical and medical cancer inpatients, and to use a venous thromboembolism risk scoring system (mainly the Khorana score) to stratify individual patient risk among ambulatory patients receiving systemic therapy [15,17,91,92]. The guidelines also suggest evaluating the risk of each patient at the moment of hospital discharge and continuing medical prophylaxis of those patients at high risk, even if the optimal long-term approach has not yet been defined [97,101,102]. However, the Khorana score, as well as other risk assessment models suggested for the stratification of individual venous thromboembolism risk, do not specifically target gynecological tumors, suggesting the need to consider every patient’s profile besides their assessment for venous thromboembolism as evaluated via the risk assessment models, applying a gestaltic approach [73,108,112,113,114,115].

Evidence from the literature suggests that there is still significant heterogeneity in daily clinical practice for prophylactic protocols of venous thromboembolism in oncological patients [83]. In gynecologic surgical patients, thromboprophylaxis with either unfractionated heparin or low-molecular-weight heparin is recommended only in cases of major surgery; these measures should begin before surgery and continue for at least 7–10 days, including extending up to 30 days in high-risk patients. Otherwise, different studies suggest that extended prophylaxis is unnecessary for patients undergoing minimally invasive interventions [105,106,107].

## 10. Conclusions

A deeper understanding of the pathophysiology of venous thromboembolism in gynecological cancers may help in patient risk stratification, to identify which patients may most benefit from different types and durations of prophylaxis.

However, the current knowledge about the prevention of venous thromboembolism in gynecological malignancy is largely based on studies on solid cancers in general. Only a few smaller studies targeting gynecological malignancy are available, providing moderate-quality evidence on this specific issue. Hence, further large and well-designed studies in this area are needed. In the meantime, clinicians should continue to combine current guidelines with a multidisciplinary team approach to ensure that these complex patients receive the best evidence-based approach to venous thromboembolism prevention and management.

## Figures and Tables

**Table 1:** Venous thromboembolism risk factors in gynecological cancer population.

Risk Factor	OR (95% CI)	Ref.
Age > 60 years	OR: 1.03 (95% CI: 1.00–1.05)	[42]
Body mass index > 30	OR: 1.92 (95% CI: 1.03–3.57)	[43]
	OR: 1.31 (95% CI: 1.20–1.50)	[42]
Tumor stage	OR: 1.11 (95% CI: 0.10–5.50)	[42]
Surgery	OR: 0.36 (95% CI: 0.30–1.00)	[42]
Operation time	OR: 1.00 (95% CI: 0.90–1.2)	
Chemotherapy	OR: 3.73 (95% CI: 1.90–7.32)	[43]
D-dimer	OR: 1.00 (95% CI: 1.00–1.05)	[42]
Hemoglobin < 11.5	OR: 2.56 (95% CI: 1.41–4.67)	[43]
Cervical cancer:		[41]
● Size ≥ 50 mm	OR: 6.46 (95% CI: 1.54–44.0)	
● Stage IV	OR: 7.62 (95% CI: 1.87–30.5)	
Ovarian cancer:		[44]
● Clear cell histology	OR: 2.8 (95% CI: 0.6–12.6)	
● Stage III and IV	OR: 3.7 (95% CI: 1.1–13.2)	
● Grade 2 and 3	OR: 2.3 (95% CI: 1.3–4.13)	
● Medium/high surgical complexity	OR: 3.2 (95% CI: 0.8–12.5)	
Endometrial cancer:		[45]
● Non-endometrioid histology	OR: 1.9 (95% CI: 1.3–2.8)	
● Stage III and IV	OR: 2.3 (95% CI: 1.5–3.4)	
● Laparotomy (laparoscopy as reference)	OR: 1.6 (95% CI: 1.05–2.34)	

OR: odds ratio.

**Table 3:** Main guidelines’ recommendations for venous thromboembolism prevention in cancer patients.

Association	Hospitalized Patients	Surgical Patients	Ambulatory Outpatients on Systemic Therapy	Patients Undergoing Central Venous Catheter
AIOM 2021 [91]	Low-molecular-weight heparin or unfractionated heparin or fondaparinux in all patients	Low-molecular-weight heparin: mechanical methods suggested but should be used as monotherapy only if prophylaxis is contraindicated Fondaparinux or unfractionated heparin can be considered in major cancer surgery (pelvic, abdominal) Duration: started preoperatively, last for 7 days, and extended to 4 weeks in high-risk groups	Low-molecular-weight heparin indicated only in high-risk patients (Khorana Score > 2)	Not indicated as routine prophylaxis
ASCO 2020 [15]	Low-molecular-weight heparin or unfractionated heparin or fondaparinux in all patients After discharge, prophylaxis should be continued in high-risk patients (Khorana score > 2)	Low-molecular-weight heparin: mechanical methods can help prophylaxis but should be used as monotherapy only if medical prophylaxis contraindicated Fondaparinux or unfractionated heparin can be considered in major cancer surgery (pelvic, abdominal) Duration: started preoperatively, last for 7 days, extended to 4 weeks in high-risk groups	Low-molecular-weight heparin indicated only in high-risk patients (Khorana Score > 2)	Not reported
NCCN 2020 [92]	Low-molecular-weight heparin or unfractionated heparin or fondaparinux Prophylaxis should be continued in at-risk medical patients (Khorana score > 2) after discharge for up to 3–6 months	Low-molecular-weight heparin, unfractionated heparin and fondaparinux in major abdominal or pelvic surgery Duration: 4 weeks after admission	Indicated up to 6 months only in high-risk patients (Khorana score > 2)	Not indicated as routine prophylaxis
ESMO 2023 [17]	Low-molecular-weight heparin represents the agent of choice	Low-molecular-weight heparin or unfractionated heparin is standard of care in surgical patients with a high risk of VTE and a low risk of bleeding Low-molecular-weight heparin once daily and unfractionated heparin three-times daily have comparable efficacy Low-molecular-weight heparin has a lower risk of heparin-induced thrombocytopenia and a more convenient administration schedule	Apixaban, rivaroxaban, or low-molecular-weight heparin may be considered for a maximum of 6 months only in high-risk patients (Khorana score > 2; the risk can individually be calculated with the Vienna-CATS nomogram score and the COMPASS-CAT score)	Compared with no prophylaxis, low-molecular-weight heparin may reduce catheter-related thrombosis without increasing the risk of bleeding. However, the absolute effect is low (38 fewer events per 1000) and the burden of injection is considerable
ITAC 2022 [16]	Low-molecular-weight heparin or fondaparinux in patients with reduced mobility	Low-molecular-weight heparin in major abdominal or pelvic surgery (laparotomy or laparoscopy) in patients without a high-bleeding risk Duration: 4 weeks	Direct oral anticoagulants only in high-risk patients (Khorana Score, COMPASS-CAT score)	Not indicated as routine prophylaxis

AIOM: Italian Association of Medical Oncology; ASCO: American Society of Clinical Oncology; NCCN: National Comprehensive Cancer Network; ESMO: European Society of Medical Oncology; ITAC: International Initiative on Thrombosis and Cancer.
